# Gene Therapy for Recurrent Laryngeal Nerve Injury

**DOI:** 10.3390/genes9070316

**Published:** 2018-06-25

**Authors:** Koji Araki, Hiroshi Suzuki, Kosuke Uno, Masayuki Tomifuji, Akihiro Shiotani

**Affiliations:** Department of Otolaryngology-Head & Neck Surgery, National Defense Medical College, Saitama 3598513, Japan; baggio2625164@yahoo.co.jp (H.S.); unotaroo@yahoo.co.jp (K.U.); tomifuji@ndmc.ac.jp (M.T.); ashiotan@ndmc.ac.jp (A.S.)

**Keywords:** gene therapy, recurrent laryngeal nerve, vocal fold, misdirected reinnervation, neurotrophic factor, adenovirus, adeno-associated virus, Sendai virus, non-viral gene delivery, electroporative gene delivery

## Abstract

Recurrent laryngeal nerve (RLN) injury has considerable clinical implications, including voice and swallowing dysfunction, which may considerably impair the patient’s quality of life. Recovery of vocal fold movement is an essential novel treatment option for RLN injury. The potential of gene therapy for addressing this issue is highly promising. The target sites for RLN gene therapy are the central nervous system, nerve fibers, laryngeal muscles, and vocal cord mucosa. Gene transduction has been reported in each site using viral or non-viral methods. The major issues ensuing after RLN injury are loss of motoneurons in the nucleus ambiguus, degeneration and poor regeneration of nerve fibers and motor end plates, and laryngeal muscle atrophy. Gene therapy using neurotrophic factors has been assessed for most of these issues, and its efficacy has been reported. Another important matter for functional vocal fold movement recovery is misdirected regeneration, in which the wrong neurons may innervate other laryngeal muscles, where even if innervation is reestablished, proper motor function is not restored. Novel strategies involving gene therapy bear promise for overcoming this issue and further investigations are underway.

## 1. Introduction

**Figure 1:**
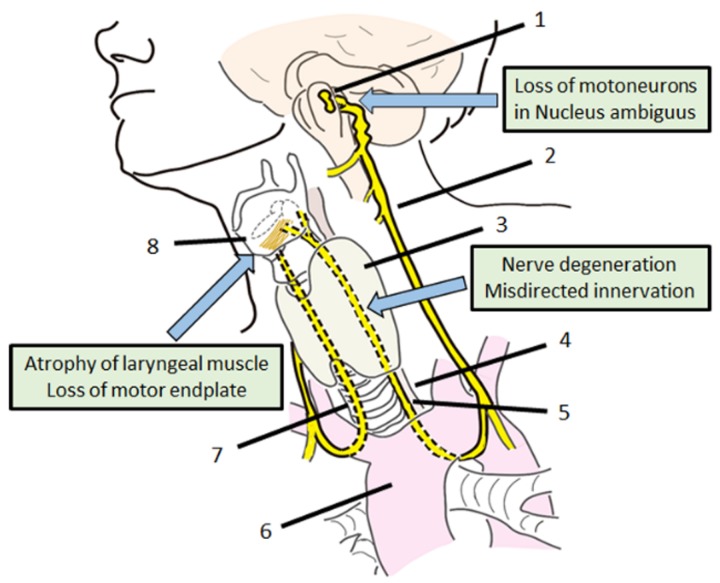
Anatomy and problems of recurrent laryngeal nerve regeneration. 1: nucleus ambiguous, 2: vagus nerve, 3: thyroid, 4: esophagus, 5: left recurrent laryngeal nerve, 6: aortic arch, 7: right recurrent laryngeal nerve, 8: vocal cord.

The recurrent laryngeal nerve (RLN), which carries motor, sensory, and parasympathetic fibers to the larynx [1], is a branch arising from the vagus nerve (VN), also known as the 10th cranial nerve. The left RLN hooks around the aortic arch and the right RLN loops below the subclavian artery. Both RLN ascends toward the transesophageal groove before entering the larynx [2] (Figure 1). The RLN provides sensory and motor innervation to the intrinsic muscles of the larynx, except to the cricothyroid muscles [2]. As the course of the left RLN follows a longer route, the incidence of left side injury has a higher rate than that of the right side [3]. 

RLN injury results in many clinical problems which may seriously impair the patient’s quality of life. The symptoms of unilateral RLN injury are hoarseness and/or dysphagia, caused by vocal fold paralysis and insufficient glottal closure. Some patients experience severe aspiration and breathy hoarseness due to widely dilated vocal fold fixation, resulting in recurrent aspiration pneumonia, exceedingly short phonation time and considerable loss of voice. In the case of bilateral RLN injury, the conservation of the appropriate position of the fixed vocal folds that enables the maintenance of the airway, vocal function, and swallowing is extremely difficult. To conserve voice quality, the glottal closure should be narrow, which may cause respiratory distress due to narrowing of the airways. To conserve the airway, the glottal closure should be wide, which worsens voice and swallowing function. Therefore, many patients with bilateral RLN injury need to keep tracheostomy. 

The major causes of RLN injury are idiopathic, surgical injury and invasion of malignant tumor of the thyroid, larynx, esophageal or an aortic aneurysm, tracheal intubation, upper airway infection, trauma, and systemic neuromuscular diseases [3]. The prevalence of temporary and permanent RLN injury post thyroid surgery has been estimated between 0% and 11% [4]. The surgical injury is one of a major cause of RLN injury and should have made some intervention because it is iatrogenic and easy to approach the injury site during surgery. However, reinnervation procedures of RLN have had little impact on restoring dynamic laryngeal function and are still not widely accepted as treatment options [5]. Main surgical options for the management of patients with unilateral laryngeal paralysis (vocal fold injection, thyroplasty, and arytenoid adduction) only achieve vocal fold medialization due to static changes in the vocal fold tissue or laryngeal framework, and such deficits can never be neurologically restored [6].

The failure of reinnervation after RLN injury may be attributed to multiple factors, including decreases in motor fiber density, atrophy of laryngeal muscle, loss of motoneurons in the motoneuronal nucleus (nucleus ambiguus in medulla oblongata) [5], and inappropriate or misdirected innervation by antagonistic motoneurons [7,8] (Figure 1). As described above, the RLN distribute motor fibers to the intrinsic muscles of the larynx. This means that the RLN innervates both adductor and abductor muscles of the vocal folds. Nonselective regeneration can lead to faulty innervation after nerve regeneration, in which the neurons may innervate improper laryngeal muscles (misdirected reinnervation), so that even if innervation is reestablished, a proper motor function is not restored (synkinesis) [5]. Therefore, novel adjuvant approaches to improve the regeneration in injured RLN injury have long been needed to further improve recovery of function. 

The potential of gene therapy is highly promising for the treatment of peripheral nerve injury. It can be considered as a type of drug-delivery system by transducing a gene and producing therapeutic proteins for a certain period of time with single administration. Successful gene delivery to motor neurons and to Schwann cells of peripheral nerves has been reported with various viral vectors such as Herpes simplex viral vectors and adeno-associated viral vectors (AAV) [9]. RLN injury is an ideal target for gene therapy because the lesion sites are accessible but not so easy for repeated access. 

Recent advances in neurology have led to the discovery of several neurotrophic and growth factors. These factors have regenerative and protective effects on the central nervous system and myoneural function through motoneurons, nerve fibers, motor endplates, and muscles. The impact of these factors using gene therapy is often investigated on peripheral nerve regeneration. These neurotrophic factors may also be useful in treating RLN paralysis [5]. 

The aim of this paper is to review and discuss the reported results of gene therapy for RLN injury and its current and future prospects. Treatment targets for functional recovery in RLN injury include vocal cords, laryngeal muscles, neuromuscular junctions, axons fibers of RLN, and motor neurons in nucleus ambiguus. Regeneration of nerve fibers, as well as appropriate reinnervation to overcome misdirection, is needed to achieve real functional recovery. In this paper, reports on the gene transfer and the therapeutic effect of gene therapy in each target sites were reviewed. In addition, future prospective strategies to overcome the misdirected reinnervation which is as the most important and challenging problem is discussed. 

## 2. Gene Transduction Methods for Recurrent Laryngeal Nerve Injury

As the first step of gene therapy for RLN injury, confirmation of gene transduction is necessary. The target sites for RLN gene therapy are the central nervous system to protect motor neurons, nerve fibers to enhance axonal regeneration, and laryngeal muscles to protect neuromuscular endplates and prevent muscle atrophy [5]. The vocal cord mucosa is also considered as a target for the treatment because gene transduction to vocal cord enables treatment from laryngeal muscle to central nervous system via retrograde axonal flow. The appropriate vector or methods to transduce therapeutic genes would differ from the target site. The reports on gene transduction for RLN gene therapy are reviewed as follows, classified by target site (Table 1).

### 2.1. Recurrent Laryngeal Nerve Fibers and Central Nervous System

Remote delivery of viral vectors to the central nervous system holds promise for the treatment of RLN injury. Viral vectors carrying therapeutic genes can be delivered to the central nervous system (CNS) through remote injection into the RLN. 

The current predominant vector of choice for gene therapy in the nervous system are AAV [9]. These vectors have the best safety profile of all available vectors to date. Rubin et al. reported that remote delivery of rAAV-GFP to the rat brainstem is possible through direct injection into the RLN [9]. Diffuse reporter gene (*GFP*) expression was observed in the brainstem, containing the nucleus ambiguus, at 3 and 11 weeks. At 11 weeks, *GFP* expression was seen not only within the ipsilateral nucleus ambiguus, but also outside of the nucleus ambiguus and in the contralateral side of the brainstem. The presence of actual viral DNA was demonstrated within the rat brainstem by in situ hybridization. This result suggests that the virus itself, rather than just the transgene product, is transported retrogradely and transsynaptically within the CNS [10]. 

Adenoviruses have also been used for gene therapy to the nervous system [11,12,13,14]. A few studies have reported remote delivery of an adenoviral vector to the damaged recurrent laryngeal nerve. Rubin et al. reported that remote injection of viral vectors into the RLN did not cause significant additional neuronal injury, by counting motor endplates contact [15]. Most of the virus within the brainstem, confirmed by fluorescent in situ hybridization, was seen in the region of the ipsilateral nucleus ambiguus one week after injection. However, a diffuse contralateral spread of the virus, similar to the AAV vector, was present [15]. Araki et al. also reported successful retrograde gene expression in the nucleus ambiguus in the ipsilateral side in the same animal model [16]. In contrast to previous reports, they showed that no motoneurons or axons were labeled in the nucleus ambiguus of the contralateral side four or five days after injection, by histochemistry of the reporter gene (*LacZ*), reverse transcription PCR (RT-PCR) analysis of the treatment gene, and immunohistochemistry of treatment-gene expression [16]. 

In the vagal nerve avulsion model, Saito et al. and Moro et al. reported successful adenoviral reporter gene (*LacZ*) expression by X-gal histochemistry. Therapeutic gene expression was also detected by RT-PCR and immunohistochemistry in the ipsilateral nucleus ambiguus. Infection of the vector to the contralateral nucleus ambiguus was not detected by RT–PCR or immunohistochemistry [17,18]. 

### 2.2. Laryngeal Muscles

Transducing therapeutic genes to the laryngeal muscles enables myotrophic and neurotrophic effects to prevent muscle atrophy and preserve motor endplates after RLN injury. 

A polyvinyl-based formulation of a muscle-specific non-viral vector containing the actin gene promoter, which produces high levels of muscle-specific gene expression, was assessed to transduce reporter (*LacZ*) and therapeutic gene (*IGF-1*) into paralyzed rat laryngeal musculature [19]. Four weeks after injection, intracellular deposition of reporter gene chromogen within thyroarytenoid and lateral cricothyroid muscle fibers was seen in seven of eight animals (87.5%). PCR analysis identified plasmid DNA of the therapeutic gene (*hIGF-I*) in 16 of 16 (100%) animals four weeks after injection. RT-PCR analysis detected mRNA of the therapeutic gene (*hIGF-I*) in 13 of 16 (81.3%) animals in the therapeutic treatment group four weeks after injection [19,20]. 

The biological effects of single vs multiple injection (once a week, repeated thrice) for this gene transfer method were also assessed [21]. Gene expression detected by RT-PCR for *hIGF-1* mRNA was demonstrated in 13 (81%) of 16 animals receiving single injections and 14 (100%) of 14 animals receiving multiple injections at four weeks after first injection. Quantitative RT-PCR for RT-PCR-positive animals showed no significant difference in transcript copies when comparing the two groups [21]. Considering the invasion of procedure, multiple injections seem to be not so favorable. 

The effects of timing for gene delivery were also assessed in the same model to evaluate delayed treatment after RLN injury [22]. The effects of non-viral gene transfer for the delivery of *hIGF-1* were examined in rats treated immediately following RLN transection and repair and in rats receiving delayed treatment scheduled 30 days after injury. Gene transfer efficiency was determined using PCR and RT-PCR. Ninety days after RLN sectioning, repair, and injection, PCR analysis identified treatment-gene (*IGF-1*) plasmid DNA in seven (53.8%) of 13 animals in the immediate-injection group and nine (69.2%) of 13 animals in the delayed-injection group. Using RT-PCR analysis, treatment-gene (*IGF-1*) mRNA was detected in three (23.1%) of 13 animals in the immediate-injection group and four (30.8%) of 13 animals in the delayed-injection group. These results demonstrated that delayed injection of the therapeutic gene may benefit the treatment for RLN injury [22]. 

Electroporative (EP) gene delivery is a method whereby cells are exposed to a brief, high-intensity electric field that induces temporary pores in the plasma membrane. The injected polyanion DNA is delivered into the cell through the pores by electrophoretic force. The efficiency of in vivo EP gene transfection was assessed in laryngeal muscle with five different conditions of high and low field voltage [23]. The condition of high and low voltage followed by low voltage with opposite polarity showed the best result, with less interindividual variability and an extended expression period. With the exception of repeated high voltage sequences, EP parameters were not likely to induce cell injury or inflammation. These results demonstrated that EP gene delivery can be used as a novel gene transduction method in laryngeal muscle with high transfection rates and limited tissue trauma [23]. 

### 2.3. Vocal Cord Mucosa

The Sendai virus (SeV) is a member of the paramyxovirus family, and it is an enveloped virus with a non-segmented, negative-sense RNA genome. The SeV has a strong affinity to the airway epithelium because wildtype SeV causes respiratory tract infection in rodents. Its replication and gene expression are driven by a viral RNA-dependent RNA polymerase strictly within the cytoplasm [24]. The SeV has unique features as a safe vector, with no pathogenicity in humans, as the RNA genome does not undergo a DNA phase. There is no risk of unwanted integration of foreign sequences into chromosomal DNA, which is associated with other conventional vectors, such as the lentivirus. Furthermore, high gene-transduction efficiencies have been reported in many tissues, including the airway epithelial [25,26], inner ear [27], muscle [28] and neural [29] tissues. The SeV infects airway epithelial cells very efficiently; therefore, the SeV vector has been tested as a potential gene-transfer vector for the treatment of cystic fibrosis [30,31]. The SeV vector can carry genes of up to at least 5 kilobases (kb) in size, and it requires less than five minutes of vector-cell contact time to introduce genes into cells [32]. Transduction to the laryngeal or tracheal epithelium by SeV was investigated by Mizokami et al. [33]. Delivery by spray inhalation of the SeV vector resulted in significant and persistent expression of the reporter gene in normal laryngotracheal epithelium compared to vector injection into the vocal cord. Transgenic SeV-mediated expression was maximal 3 days after inhalation, decreased over time, but remained detectable for 14 days after administration. No serious side effects were observed in the larynx or trachea. Efficient SeV-mediated transgene expression was also observed in the injured mucosa at the levels of the trachea, cricoid cartilage, and vocal cords. Successful SeV-mediated transgene expression in normal tissue and in the injured mucosa of the larynx was demonstrated. The finding that the spray inhalation method showed better transduction sufficiency compared to injection is promising, as it introduces the possibility of developing simple inhalation methods for gene transduction [33]. 

## 3. Gene Therapy for Recurrent Laryngeal Nerve Injury

The neurotrophic and growth factors including nerve growth factor (NGF), brain-derived neurotrophic factor (BDNF), glial cell line-derived neurotrophic factor (GDNF), fibroblast growth factor (FGF), ciliary neurotrophic factor (CNTF), vascular endothelial growth factor (VEGF), and insulin-like growth factor I (IGF-I) have potential for peripheral nerve regeneration. The impact of gene therapy transducing these factors is often investigated. For example, one report compared the effect of six factors on axon regeneration of the sciatic nerve using lentiviral gene therapy [34]. Three of the six neurotrophic factors (BDNF, GDNF, and NGF) showed enhanced modality specific axon outgrowth after autograft-based repair combined with gene therapy. The reports of gene therapy for RLN injury are reviewed as follows classified by main target site of the lesion. (Table 2).

### 3.1. Prevention of Laryngeal Muscle Atrophy and Preservation of Nerve Endplates

Shiotani et al. [19] developed a rat laryngeal paralysis model sutured after a 1 cm gap to assess novel gene transfer strategies. Using this model, the *IGF-I* gene was introduced into paralyzed rat laryngeal muscle. A muscle-specific non-viral vector containing the a-actin promoter and *IGF-I* gene was used in formulation with a polyvinyl-based delivery system and injected into paralyzed adult rat laryngeal muscle as described in Section 3.2. Twenty-eight days after a single injection, *IGF-I*-transfected animals presented a significant increase in muscle fiber diameter, motor endplate length, and percentage of endplates with nerve contact when compared to controls [19,20]. 

Myosin heavy chain (MHC) composition was analyzed after *IGF-I* gene transfer in denervated rat laryngeal muscle to determine whether the myotrophic activity of IGF-I promotes restoration of normal MHC composition after nerve injury [35]. MHC composition in denervated laryngeal muscle was characterized by a decrease in type IIB and IIL and up-regulation of IIA/IIX. Compared to controls, IGF-I-treated animals demonstrated a significant increase in expression of type IIB and IIL and a significant decrease in expression of type IIA/X. These findings suggest that the myotrophic effect of *IGF-I* gene transfer results in normalization of MHC composition in denervated muscle, with suppression of type IIA/X MHC and promotion of type IIL expression [35]. 

Comparison of the effect of single vs multiple injections of the same treatment was performed [21]. Higher gene expression rate was detected by RT-PCR for *IGF-I* mRNA in animals receiving multiple injections when compared to animals receiving a single injection (100% vs. 81%). Compared to controls, *IGF-I*-transfected animals in both the single- and multiple-injection groups had a significant increase in the lesser diameter of muscle fiber, a significant decrease in motor endplate length, and a significant increase in the percentage of endplates with nerve contact. Although the percentage of denervated muscles demonstrating IGF-I expression was increased following multiple injections, no difference was observed in the biological response compared to that in the single-injection treatment groups [21]. 

In the clinical setting, the treatment of RLN injury is not always performed immediately after onset. Watchful waiting is employed for a few weeks to months, as spontaneous recovery might be achieved in many cases. The animals in the experiments described above were treated immediately after RLN injury. Moreover, the difference of effect was compared in rats treated immediately and in rats receiving delayed treatment 30 days after injury [22]. Compared to reinnervated untreated control samples, both early and delayed *IGF-I* transfer resulted in significant increase in muscle fiber diameter. Motor endplate length was significantly decreased, and nerve/motor endplate contact was significantly increased following delayed gene transfer but not after immediate treatment. The authors concluded that delayed *IGF-I* gene transfer, delivered by a single intramuscular injection, would enhance the process of muscle reinnervation [22]. 

Rubin et al. demonstrated the ability to enhance nerve regeneration in a rat RLN crush model by an AAV vector carrying a zinc-finger protein (*ZFP*) transcription factor, which stimulates endogenous IGF-I production [36]. The AAV vector was directly injected into the crushed RLN. The difference between the percentages of nerve endplate contact on the crushed and uncrushed sides was statistically significantly lower in the experimental group one week after injection. The visual analogue scale score that evaluated vocal fold motion one week after injection was significantly higher in the experimental group. The authors concluded that treatment using an AAV vector demonstrated a neurotrophic effect when injected into the crushed RLN [36]. 

Sakowski et al. also demonstrated the efficacy of *ZFP* gene-carrying adenovirus (Ad-p65), which induces expression of VEGF in rat RLN crush model [37]. At seven days post-crush, rats receiving the Ad-p65 construct had a significantly increased percentage of nerve endplate contact compared to controls. An enhanced restoration of nerve-endplate contact in rats undergoing RLN nerve-crush injury was noted after Ad-p65 injection [37]. 

### 3.2. Prevention of Motoneuron Loss in the Central Nervous System

One of the main problems in the treatment of laryngeal paralysis is motoneuron loss in the nucleus ambiguus, the motoneuronal nucleus of the RLN. Motoneuron loss results in irreversible injury and the regeneration of the neural system after motoneuron loss is challenging. To assess the potential of gene therapy for motoneuron loss reversal after vagal or recurrent laryngeal nerve injury, Saito et al. demonstrated the neuroprotective effects of an adenoviral vector encoding GDNF on lesioned adult rat motoneurons in the nucleus ambiguus [17]. Vagal nerve avulsion is the model introducing marked atrophy and loss of motoneurons in the nucleus ambiguus. Avulsion and inoculation with treatment vector prevented the loss of lesioned motoneurons in the nucleus ambiguus. Immunoreactivity of the choline acetyltransferase (ChAT), which is the most specific indicator for monitoring the functional state of cholinergic neurons, was ameliorated, and the activity of nitric oxide synthase (NOS), which plays a significant role in the initiation of adult motoneuron loss, was also suppressed in these neurons [17]. 

Moro et al. examined the synergistic neuroprotective effects of adenoviral gene transfer of *BDNF* and/or *GDNF* in the same animal model [18]. The treatment with GDNF or BDNF significantly prevented the loss of motoneurons compared to controls. The protective effect of BDNF was greater than that of GDNF. Combined treatment with BDNF and GDNF acted synergistically and a significantly larger number of motoneurons in the nucleus ambiguus was preserved as compared to either BDNF or GDNF treatment. Treatment with BDNF and/or GDNF after avulsion also suppressed the activity of NOS in lesioned motoneurons in the nucleus ambiguus. These results indicate that adenovirus-mediated *BDNF* and/or *GDNF* gene transfer may prevent the degeneration of motoneurons after either vagal nerve injury or recurrent laryngeal nerve injury [18].

### 3.3. Regeneration of Nerve Fibers and Neurofunctional Recovery

Another major complication after RLN injury is the degeneration and poor regeneration of nerve fibers. There are few reports assessing neurofunctional and histological recovery after RLN gene therapy. 

Araki et al. demonstrated functional and histological recovery after adenoviral *GDNF* gene transfer directly injected into the crushed site of rat RLN [16]. Animals treated with adenoviral *GDNF* displayed significantly improved motor nerve conduction velocity (MNCV) of the RLN. GDNF-treated animals showed near normal MNCV 4 weeks after treatment. These animals showed significantly larger axonal diameter and improved remyelination in the crushed RLN site compared to controls. Adenoviral *GDNF* gene transfer strongly promoted histological regeneration and neurofunctional recovery after RLN injury [16]. 

A second report utilized remotely delivered adenoviral vectors (Ad-p65) encoding engineered ZFP transcription factors, which induce expression of VEGF [37]. Ad-p65 transfection of primary motoneurons in vitro results in VEGF variant expression and a significant increase in axon outgrowth in these cells. Injection of Ad-p65 after RLN crush accelerated the return of vocal fold mobility and preserve the nerve-endplate contacts in the thyroarytenoid muscle. Ad-p65 induced VEGF expression and enhanced nerve regeneration [37]. 

### 3.4. Functional Recovery of the Larynx

The endpoint of these studies was the recovery of normal vocal fold movement, which is synchronized with breathing, swallowing, and phonation. Araki et al. published the first report of vocal fold movement recovery using gene therapy in a rat RLN crush model [16]. They directly injected GDNF encoding adenovirus into the crush site of the RLN. The number of rats that apparently recovered vocal fold movement was 4/4 (100%) two weeks and 4/4 (100%) four weeks after injection in adenoviral GDNF-treated animals. The recovery rate was significantly higher in GDNF-treated animals than in controls [16]. 

Sakowski et al. reported the effect of Ad-p65, which induces the expression of VEGF, in a rat RLN crush model [37]. Significant increases in vocal fold motion upon direct laryngoscopy occurred in Ad-p65-treated rats compared to in control animals seven days after injection. This gene therapy restored vocal fold motion earlier than it occurred in untreated rats [37]. 

Rubin et al. also reported vocal fold motion recovery in a rat RLN crush model after adeno-associated viral ZFP gene therapy, which stimulates endogenous IGF-1 production. The difference in the visual analogue scale score seven days after injection between the experimental and control groups was statistically significant [36]. 

All of these studies presented functional recovery of vocal fold motion in RLN-crush models. In the crush peripheral nerve model, the injury level is not as severe and does not result in loss of motoneurons in the nucleus ambiguus [38]. In the crush injury model, the misdirected regeneration is not as affected for functional recovery when compared to in the axotomy model, because the integrity of the nerve is retained. Though these gene therapies have been proven effective for moderate RLN injury, the most important issue for functional recovery in RLN injury, misdirected regeneration, still remains to be overcome.

## 4. Limitations and Problems of Previous Research for Recurrent Laryngeal Nerve Injury

As described above, it has been demonstrated that gene therapy is effective in preventing laryngeal muscle atrophy and motoneuron loss, preserving nerve endplates, regenerating nerve fibers, and enhancing neurofunctional recovery in animal RLN injury models. Although the researchers are limited in this field, the results were decent to consider and expect clinical application. However, from the viewpoint of future clinical application, some problems that require further study are recognized. Particularly, each research used various animal models, different gene transduction methods and various therapeutic factors made it difficult to compare the results of each study. It is necessary to identify optimal gene introduction methods and therapeutic factors for each target sites from multiple methods and therapeutic factors in the same condition. 

Although some report mentioned that there was no histological damage and immunological response as an associated disorder with gene therapy, no studies focusing on the side effect had been reported. The studies demonstrated the valid gene transduction by the remote delivery of AAV or Adenovirus vectors to the central nervous system, they also showed the limitations [10,15]. They concluded that attempts to treat focused areas of the CNS, such as the nucleus ambiguus, will be limited by the potential side effects of diffuse delivery. The limited duration of gene expression of adenovirus seems to be favorable characteristics for RLN injury because temporary gene expression is suitable for short-term treatment, one mechanism of short-term transgene expression is supposed to be caused by an immune response to the transduced cells. That is the reason that adenoviral vectors have been abandoned by most researchers due to their toxicity and immunogenicity. Further preclinical study to increase the evidence level not only in terms of efficacy but also in terms of safety is necessary for clinical application. 

## 5. Future Directions of Gene Therapy for Recurrent Laryngeal Nerve Injury

To achieve functional recovery of vocal fold movement after severe RLN injury, the prevention of misdirection must be achieved. One strategy to recover vocal fold motion is targeting axon regeneration enhancement exclusively in the adductor muscle. As the only abductor muscle of the vocal fold is the posterior cricoarytenoid (PCA) muscle, prevention of axon regeneration to the PCA results in a strengthening of the adductive function of the vocal fold and recovery of vocal fold motion. 

A few studies have reported the difference in expression of neurotrophic factors in laryngeal muscles. Vega-Cordova et al. reported that BDNF expression was unchanged in the thyroarytenoid muscle (TA) but was diminished in both PCA muscles three days and six weeks after injury, returning to near-normal levels four months after injury [39]. Halum et al. compared the differences in gene expression of five well-characterized NFs between the PCA muscle and the adductor complex after RLN or VN transection injuries [40]. Notable differences three days after injury included greater GDNF expression from the PCA muscle relative to the adductor after VN injury, and greater IGF-1, CNTF, and VEGF expression from the PCA muscle relative to the adductor after RLN injury. One month after injury, adductor BDNF expression was greater than PCA BDNF expression in both the VN and RLN injury groups, and adductor VEGF expression was greater than PCA VEGF expression in the RLN injury group [40]. Hernandez-Morato et al. reported the expression of GDNF in the abductor and adductor muscles in the rat transection and anastomosis RLN model [41]. Significant upregulation of GDNF was observed until 14 days after RLN injury. The highest level of GDNF expression was reached at different times in the PCA, lateral thyroarytenoid (LTA), and medial thyroarytenoid (MTA) muscles. These expression peaks correlated with the timing of reinnervation observed on immunohistochemistry, where PCA was reinnervated first, followed by MTA and LTA [41]. 

Such differences in the expression of neurotrophic factors in each laryngeal muscle can be a therapeutic target for promoting appropriate reinnervation of the RLN. Hernandez-Morato et al. reported the effect of the anti-GDNF antibody on RLN reinnervation [42]. After injection of the anti-GDNF antibody into the PCA, vocal fold function was improved as compared to controls. Early arriving axons bypass the PCA and enter the LTA and later arriving axons innervate the PCA and MTA. Anti-GDNF antibody injection into the PCA influences the pattern of reinnervation and may result in less synkinetic, more functional innervation [42]. Similarly, in addition to neurotrophic factors, vincristine, an anti-cancer agent, has been used to block PCA from synkinetic reinnervation and improve laryngeal adductor functional recovery [43]. 

The strategy of controlling the expression of neurotrophic factors to improve reinnervation to only adductor muscles might be suitable for gene therapy. Gene transduction into laryngeal muscles has been reported and gene therapy that enables long-term effective periods by a single administration has great advantages. 

Another strategy for functional recovery of the vocal fold is the reduction of misdirected reinnervation between motor nerve and other fibers. The RLN consists of motor fibers also in addition to sensory and autonomic nerve fibers. Misdirection between motor and sensory or autonomic fibers is supposed to be one of the major causes of functional recovery failure. We have reported the value of a novel PGA-collagen tube on RLN regeneration as a scaffold for drug or vector administration in RLN regeneration [44]. An agent that inhibits the expansion of sensory axons is inserted into this tube and bridged to the RLN after transection. Our data demonstrated good vocal fold motion recovery rate (more than 40%) in experiments using rats ([45], unpublished data). 

The combination of these strategies might overcome the problem of misdirected regeneration after RLN injury and further studies are necessary. These strategies might have great potential for clinical application for laryngeal paralysis as well as other forms of peripheral motor nerve paralysis. 

## 6. Conclusions

RLN injury has considerable clinical implications, including voice and swallowing dysfunction, which may seriously impair the patient’s quality of life. The potential of gene therapy for addressing this issue is highly promising. The target sites for RLN gene therapy are the central nervous system to protect motoneurons, nerve fibers to enhance axonal regeneration, and laryngeal muscle and vocal cord mucosa to protect neuromuscular endplates and prevent muscle atrophy. Gene therapy has been employed for most of these issues, and its efficacy has been assessed. Misdirected regeneration is a crucial impediment for functional vocal fold movement recovery. Novel strategies involving gene therapy bear promise for overcoming this issue and further investigations are underway. 

## Figures and Tables

**Table 1:** Gene transduction methods targeting RLN injury.

Author	Target Site	Animal Model	Administration Method	Vector or Method	Confirmed Gene	Result
Rubin [9]	RLN ^1^ and Brainstem	Normal	Nerve injection	AAV ^2^	*GFP*	Positive at 11 weeks Both and outside NAs ^5^
Rubin [14]	RLN ^1^ and Brainstem	RLN ^1^ crush	Nerve injection	AdV ^3^	Viral DNA	Positive at 1 week Both NAs ^5^
Araki [15]	RLN ^1^ and Brainstem	RLN ^1^ crush	Nerve injection	AdV ^3^	*LacZ* *hGDNF*	Positive at 4 or 5 days Ipsilateral NA^5^
Saito [16] Moro [17]	Brainstem	Vagal nerve avulsion	Inoculation	AdV ^3^	*LacZ* *hGDNF*	Positive at 4 or 5 days Ipsilateral NA ^5^
Shiotani [18,20] Flint [19] Nakagawa [21]	Laryngeal muscles	RLN ^1^ transection and anastomosis	Muscle injection	muscle-specific non-viral vector	*LacZ* *hIGF-1*	Positive at 90 days
Saito [22]	Laryngeal muscles	Normal	Muscle injection	Electroporative gene delivery (Plasmid)	*EGFP*	Positive at 8 weeks
Mizokami [32]	Vocal cord mucosa	Normal	Spray	SeV ^4^	*GFP* *LacZ*	Positive at 14 days

RLN: recurrent laryngeal nerve, ^2^ AAV: adeno-associated viral vector, ^3^ AdV: adeno viral vector, ^4^ SeV: Sendai virus vector, ^5^ NA: nucleus ambiguus.

**Table 2:** Gene therapy targeting RLN disorder.

Author	Target Lesion	Animal Model	Vector or Method	Treatment Gene	Result
Shiotani [19,21] Flint [20,35] Nakagawa [22]	Laryngeal muscle atrophy Nerve endplate	RLN ^1^ transection and anastomosis	muscle-specific non-viral vector	*hIGF-1*	Increases in muscle fiber diameter, motor endplate length, and PEC ^4^ Normalization of MHC ^5^ composition
Saito [17]	Motoneuron loss	Vagal nerve avulsion	AdV ^2^	*hGDNF*	Prevention in motoneuron loss in NA ^6^
Moro [18]	Motoneuron loss	Vagal nerve avulsion	AdV ^2^	*hBDNF* *hGDNF*	Synergistic prevention of motoneuron loss in NA
Araki [16]	Neurofunctional recovery Vocal fold motion	RLN ^1^ crush	AdV ^2^	*hGDNF*	Improved MNCV ^7^ Larger axonal diameter Improved remyelination Better recovery of vocal fold motion
Rubin [36]	Nerve endplate Vocal fold motion	RLN ^1^ crush	AAV ^3^	*IGF-1*	Increases in PEC ^4^ Better recovery of vocal fold motion
Sakowski [37]	Nerve endplate Vocal fold motion	RLN ^1^ crush	AdV ^2^	*VEGF*	Increases in PEC ^4^ Better recovery of vocal fold motion

^1^ RLN: recurrent laryngeal nerve, ^2^ AdV: adeno viral vector, ^3^ AAV: adeno-associated viral vector, ^4^ PEC: percentage of endplates with nerve contact, ^5^ MHC: myosin heavy chain, ^6^ NA: nucleus ambiguous, ^7^ MNCV: motor nerve conduction velocity.
